# The Effects of the Ukrainian Conflict on Oncological Care: The Latest State of the Art

**DOI:** 10.3390/healthcare11030283

**Published:** 2023-01-17

**Authors:** Emma Altobelli, Paolo Matteo Angeletti, Giovanni Farello, Reimondo Petrocelli

**Affiliations:** 1 Department of Life, Public Health and Environmental Sciences, University of L’Aquila, 67100 L’Aquila, Italy; 2 Cardiac Surgical Intensive Care Unit, Giuseppe Mazzini Hospital, 64100 Teramo, Italy; 3 Public Health Unit, ASREM, 86100 Campobasso, Italy

**Keywords:** health, randomized clinical trials, RCT, cancers, war, Ukraine

## Abstract

Background: The COVID-19 pandemic has dramatically affected all aspects of the patient’s pathway to cancer diagnosis and subsequent treatment. Our main objective was to evaluate the status of cancer trials in Ukraine as of September 2022. Methods: Initially, we examined with a narrative review the state of breast, colorectal, and cervical cancer population-based screening. Subsequently, we assessed each trial status for the years 2021 and 2022. Results: Estimates of participation in breast and cervical cancer screening are different from region to region. Moreover, regarding cervical cancer screening, extremely different participation estimates were reported: 73% in 2003 vs. <10% 2020. Our data show that from 2014 to 2020, despite the pandemic, cancer trials in Ukraine significantly increased from 27 to 44. In 2021 no trials were completed; in fact, we observed that out of 41 trials, 8 were active not recruiting, 33 were recruiting, and 0 were completed or terminated. In 2022 in Ukraine, for oncological pathologies, only 3 trials were registered, while in 2021, 41 trials were registered. The suspension of trials regarded above all concern hematological tissue (66.7%) and the genitourinary tract (60%). Conclusions: Our work has highlighted how the areas most affected by the conflict present criticalities in oncological care.

## 1. Introduction

Cancers are a major contributor to disease burden worldwide, and projections forecast that global cancer burden will continue to grow for at least the next two decades [1,2,3,4]. There is a need for reducing cancer burden by one third and for reducing premature mortality from noncommunicable diseases [NCDs] through prevention and treatment [5]. It is known that population-based screening of breast, colorectal, and cervical cancer are an important secondary prevention means, they have been slowed down globally because of the priorities required by the “pandemic”. Therefore, COVID-19 disease has affected in the worldwide all aspects of the patient’s pathway to cancer diagnosis and subsequent treatment [6]. It is important to underline that non-communicable diseases in Ukraine represent a total of 80% of mortality and, in the age group between 30 and 69, they represent the leading cause of death. Before the hostilities with Russia, about 13,000 new diagnoses of cancer were diagnosed in Ukraine [7]. The development of the conflict has led to the displacement of a large number of patients to western regions and to foreign countries [8]. The impact of conflicts on the care of people with cancer has highlighted how there is a fragmentation due to the inability of humanitarian aid to cover all needs and the excessive burden that is created in the arrival countries of refugees [8]. The outbreak of the war did not catch regulatory agencies unprepared, even in light of the recent pandemic, which made it possible to make follow-ups more flexible. In particular, patients were reallocated in Europe, where possible, trying to keep the quality of information high [9].

The clinical trial is a fundamental tool for oncologic therapy. Today, free consultation of protocols, funders, and sites where the research is conducted is possible through the clinicaltrial.gov site. The clinicaltrial.gov, managed by the U.S. National Library of Medicine currently records more than 430,000 studies for 222 nations [10]. From 2000 to 2020, the trials steadily increased from 2786 per year to 671,228 per year [11].

A study has shown that about 80% of participants in clinical trials belong to high-income countries [12], while 70% of cancer deaths occur in low and medium-income countries [13]. The choice to conduct a clinical trial in a low-income country involves lower overall costs and it may represent an opportunity to care for the local population. Ukraine is a medium-low-income country and before the Russian invasion, Ukraine was considered an optimal destination for clinical trials. It has, in fact, become an attractive country for companies wishing to carry out clinical trials since 1996 (investigative report by the NGO Public Eye published in 2013) mainly due to its lower costs and some less demanding local legislative practices [14].

The objectives of our research are: (i) to conduct a narrative review of the literature on the art layer of the three tumors subjected to population-based screening; (ii) evaluate the development of cancer trials in Ukraine in the 2014–2022 period; and (iii) assess the impact of the conflict on active trials in Ukraine in the two-year period from 2021 to 2022, and examine the types of cancer for which the trials were most affected by the war.

## 2. Materials and Methods

Initially, a narrative review of the literature on the art layer of the three tumors subjected to population-based screening (breast, colorectal cancer and cervix) and HPV vaccination was conducted in order to highlight the pre-conflict Russian-Ukraine” situation and identify any changes that occurred during the conflict (Table 1).

Subsequently, we assessed the “state of the art” with regards to clinical trials in Ukraine. A search was conducted using the clinicaltrial.gov portal, using the terms “Cancer” and “Ukraine” as of 18 May 2022. The database was queried starting from 2014 in order to assess the number of trials registered in Ukraine since the beginning of hostilities with Russia in 2022. The resident population in Ukraine was considered, according to world bank data, and the number of active trials with respect to the resident population.

Each trial status was assessed for the years 2021 and 2022 regarding the following: active/suspended/terminated/not yet recruiting/completed/unknow status, the geographic region, the linguistic predominance, according to the last Ukrainian census [15,16] and type of tumor under study.

We also analyzed the 2021 trials status in two different moments (the first in May and the second in September 2022).

Differences between frequencies were assessed using chi-square or Fisher exact tests where appropriated.

**Figure 1:**
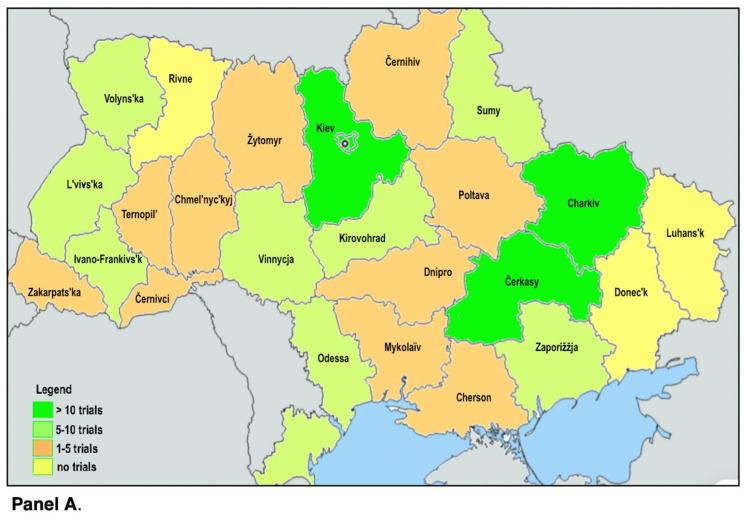

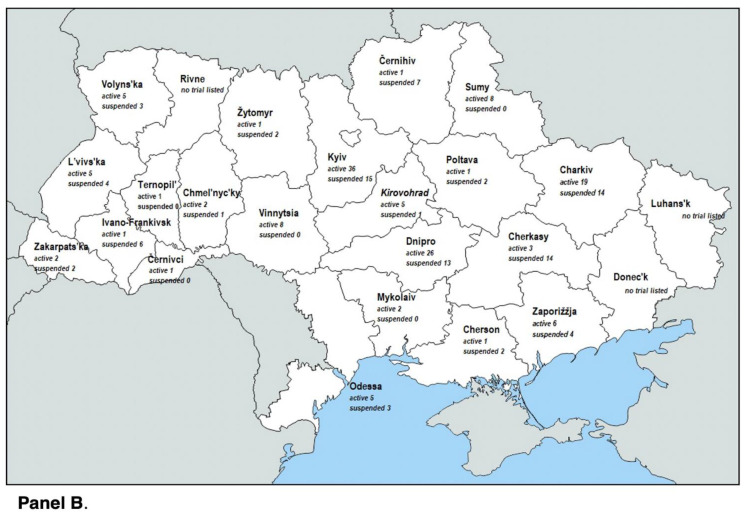
(**A**) Map according to trials grouped by following classes: >10; 5–10; 1–5. (**B**) Number of trials according to status for each Ukrainian oblast’ as of 5 May 2022.

Finally, to highlight distribution of trials among the various oblasts we have presented maps according to trials grouped by the following classes: >10; 5–10; 1–5; (Figure 1, Panel A) and number of trials according to status for each Ukrainian oblast’ (Figure 1, Panel B).

## 3. Results

As reported in Table 1, pre-conflict screening programs in Ukraine were conducted on a spontaneous basis (no population-based screening program). Estimates of participation in breast and cervical cancer screening are different from region to region. Moreover, extremely different participation estimates have been reported for over a decade regarding cervical cancer screening (73% in 2003 vs. <10%) in the WHO data 2020. Yet, with respect to the latter, there are no HPV vaccination campaigns. Primary prevention for colorectal cancer was also absent before the war.

In the period considered, the incidence of cancer in Ukraine remained constant, and on the contrary, mortality decreased (Table S1) [21].

The number of cancer trials registered in Ukraine from 2014 to 2020 has been steadily increasing, going from 27 in 2014 to 44 in 2020. In terms of incidence ratio on the total resident population, it has gone from 0.60 to values of 1.0 compared to the latest proliferation data available in 2020 (Table S1). 275 trials involved adult patients and 16 children (Table S1). It is important to underline that in 2021 no trials were completed; in fact, we observed that out of 41 trials, 8 were active and not recruiting, 33 were recruiting, and 0 were completed or terminated (Table S1). In 2022 in Ukraine, only 3 trials were registered for oncological pathologies, while 41 were registered in 2021.

The comparison between the two periods analyzed showed a reduction of not recruiting trials in May 2022 respect to September 2021 from 4% to 27% (Tables S2 and S3).

Regarding Russian and Ukrainian speaking geographical areas, and furthermore center, east, west and south regions, no statistically significant differences were found (Table 2 and Table 3).

Regarding tumor affected sites, the suspension of trials belonged above all to hematological tissue (66.7%) and the genitourinary tract (60%); in fact, there is a statistically significant difference (*p* = 0.034) (Table 4).

## 4. Discussion

With the COVID-19 pandemic, the world population has experienced high levels of stress both from a physical and psychological point of view. The Russian-Ukrainian war is now a new additional stressor. In fact, the impact of the war has repercussions on different areas of life, such as social and economic areas with the increase in the prices of energy, food, and raw materials. The healthcare system has therefore significantly been influenced. Compared to the latter, a significant fact is that only 36.08% of the Ukrainian population received the COVID-19 vaccination and of these, most received only two doses. This means a higher risk of mortality from COVID-19 [22]. Furthermore, children in conflict zones suffer the most since they do not receive necessary vaccinations [23]. In fact, it also is important to underline the Ukrainian polio outbreak from 2014 to 2017 [24] and likewise the measles outbreak in 2016 [25].

Therefore, we can stress that although Ukraine has spent most of its resources defending itself against the Russian invasion, it has now become exposed to multiple infectious diseases [23], some of which are known to be risk factors for the genesis of some cancers.

As highlighted by the review (Table 1) there is no HPV vaccination campaign: in fact, cervical cancer is the fourth most common among women [26].

It is important to underline that the development of the conflict has involved the displacement of a large number of patients to western regions and to foreign countries [8]. With regard to screening, some initiatives in refugee host countries such as the Netherlands should be highlighted [27].

The impact of conflicts on the care of people with cancer has recently been studied in the Syrian and Iraqi conflict: it has been highlighted that there is a fragmentation of care due to the inability of humanitarian aid in countries of refugee arrival [8]. Regarding cancer incidence, it should be remembered that the incidence rates of common cancers in Ukraine increased during 2003–2012, and Ryzhov et al. predicted an overall 18% increase in the number of cancer cases from 2012 to 2022 [24]_._ In addition, in Ukraine there are no organized screening programs [28,29,30]. In fact, the absence of screening programs is a feature common to several ex-Soviet countries [31], although in recent decades more progress has been made both at the diagnostic level (by adapting diagnostic techniques with those of other European countries) and organizational level [31].

Our main objective is to provide a report on the status of cancer trials in Ukraine since the beginning of the war in September 2022. Our data show that from 2014 to 2020, despite the pandemic, cancer trials in Ukraine significantly increased from 27 to 44. As van Rosmalen et al. [32] underlined, this growth trend was in line with the worldwide increase in trials. These data are also consistent compared to what is shown by the International Clinical Trials Registry of the WHO Platform (ICTRP), which shows an overall increase in trials in Ukraine from 1999 to 2021: in particular, malignant neoplasms represented 24% of all trials registered [11]. Our work has highlighted how the areas most affected by the conflict present a greater suspension of oncology trials, in particular in Phase 3, in which safety and effectiveness of a new treatment is tested against the current standard treatment. As reported by Ryzohv et al. [31], the incidence of cancer in Ukraine will increase by 14% in males and 21% in females, compared to the previous decade. In light of this, a consideration in our opinion is important. The oncological problem in Ukraine is also linked to the Chernobyl nuclear accident. In fact, Zupunski, et al. [33], showed that in the 5 years following the nuclear accident there was a significant increase in breast cancer in neighboring areas. If the war continues for much longer, the repercussions could be dramatic for people attending Ukrainian studies, for whom a trial is often the last hope against cancer.

## 5. Conclusions

Despite new therapies and increased awareness of risk factors, it is very likely that there will be a faster increase in new cancer cases in the years to come. Structural factors, such as the aging of the population, and contingent situations such as the pandemic and the war in Ukraine will play a significant role. Nonetheless, this last one did not catch regulatory agencies unprepared (even in light of the recent pandemic which made follow-ups more flexible). In fact, it was possible to reallocate patients to Western Europe, where possible, trying to keep the quality of information high [9]. However, it should be emphasized that it is currently impossible to assess the long-term impacts on drug research because it is difficult to maintain strict clinical trial protocols while hospitals are bombed.

Last but not least, there is the drama of oncological children. With the first bombings, their situation worsened further, since they were transferred from the clinics to basements, which initially, before humanitarian interventions, certainly complicated their treatments.

The future looks very uncertain indeed.

## Figures and Tables

**Table 1:** Situation on cancers early detection program in Ukraine before the 2022 war.

Cancer [17]	Incidence [17]	Mortality [17]	Data Quality [17]		Before War
			Incidence	Mortality	
Breast	11.8	8.4%	High	Medium	An opportunistic screening program is present for woman >50 years old [17]. A 2018 report showed the capacity of the system to detect an early stage of tumor is widely different among Ukraine regions from 95 % of Vinnytska Region to only 60% Luhanska [18].
Cervical	3.5%	2.5%	High	Medium	An opportunistic screening program is present. In 2020 Screening participation rates are <10% (WHO) [17], while in 2003 was reported a participation rate 73.7% in all women aged 25–64, screened every 3 y [19]. No plans for HPV vaccination were implemented [19].
Colorectal	13.1%	14.0%	High	Medium	No plans for screening were adopted. A large study showed that 50.9% with colon cancer, 60.7% with rectal and anal cancers presented at disease stages 1 and 2 [18]. The data on Crimea, Donetska, and Luganska Oblast (region) were missing in 2014 and 2015 because of political instability [20].

**Table 2:** Numbers of registered trials in 2021 according to different speaking areas and status (active/suspended) as of May and September 2022.

Status as of May and September 2022	Russian Speaking Areas	Ukrainian Speaking Areas	Total	Test
*May*				
Active	54 (37.2)	91 (62.8)	145	
Suspended	27 (34.2)	54 (65.8)	81	*X^2^ = 0.21*, *p = 0.66*
			224	
*September*				
Active	69 (38.1)	112 (61.9)	181	
Suspended	23 (28.4)	58 (71.6)	81	*X^2^ = 2.32*, *p = 0.13*
			262	

**Table 3:** Numbers of registered trials in 2021 according to different geographical areas (center, east, west, south) and status (active/suspended) as of May and September 2022.

Areas	Active	Suspended	Tot	Test
*May*				
Center	55 (65.5)	29 (34.5)	84	*X^2^ = 0.21*, *p = 0.64*
West	27 (60.0)	18 (40.0)	45	
East	20 (66.5)	12 (37.5)	32	
South	43 (68.3)	20 (31.7)	63	
*September*				
Center	59 (63.4)	34 (36.5)	93	*X^2^ = 3.45*, *p = 0.33*
West	44 (67.7)	21 (32.3)	65	
East	29 (78.3)	8 (21.7)	37	
South	49 (73.1)	18 (26.9)	67	

**Table 4:** Numbers of registered trials in 2021 according to status and diagnosis.

Tumors	Active	Suspended	Total
Gender-related	9 (100.0)	0 (0.0)	9
Gastro-intestinal	3 (60.0)	2 (40.0)	5
Hematological	2 (33.3)	4 (66.7)	6
Lung	4 (66.7)	2 (33.3)	8
Urinary tract	2 (40.0)	3 (60.0)	5
*X^2^= 15.74*, *p = 0.034*			

## Data Availability

Publicly available datasets [2] were analyzed in this study.

## Supplementary Materials

The following supporting information can be downloaded at: https://www.mdpi.com/article/10.3390/healthcare11030283/s1.
