# Supplementary material for: The Effects of the Ukrainian Conflict on Oncological Care: The Latest State of the Art
Source: Healthcare (Basel). 2023 Jan 17;11(3):283. doi: 10.3390/healthcare11030283 (PMC9914056; doi:10.3390/healthcare11030283)
Supplement: Supplementary file 1 [file healthcare-11-00283-s001.zip › healthcare-2093769-supplementary.pdf]

Supplementary Materials

Table S1. Number of oncological newly registered trials active in Ukraine from 2014 to 2022 and their main characteristics. Data about incidence and mortality are taken from Bulletin of the National Cancer Registry of Ukraine. LEGEND: ND= No data; (*) Rate per 100.000 (standardized incidence and mortality: world population).

**Year**	**Total**	**Population (milion)**	**Incidence(*)**	**Mortality(*)**	**Trial Incidence ratio**	**Trial status**						**Pediatric Trial**	**Phase**					
						Not yet recruiting	*Recruiting*	*Active, not recruiting*	*Suspended/ Terminated*	*Completed*	*Unknown status*		*1*	*2*	*3*	*4*	*Not applicable*	*Not available*
2022 sept.	3	-	ND	ND	-	-	3	-	-	-	-	-		1	2	-	-	-
2022 may	2	-	ND	ND	-	1	1	-	-	-	-	1	0	0	2	0	-	-
2021	41	44.13(*)	ND	ND	0.93	-	33	8	-	-	-	1	1	11	29	-	-	
2020	44	44.13	186.5	84.4	1.00	-	33	8	2	1	2	2	7	9	28	-	-	-
2019	40	44.39	224.2	92.9	0.90	-	22	15	3	-	-	0	1	8	28	2	1	2
2018	45	44.62	225.0	96.9	1.01	-	19	18	-	6	2	0	5	6	34	-	1	-

2017	47	44.83	224.3	100.2	1.05	-	7	20	4	16	-	3	8	13	30	-	-	-
2016	28	45	223.0	102.6	0.62	-	2	10	4	10	1	2	5	6	18	-	-	-
2015	28	45.15	225.4	103.3	0.62	-	3	10	1	12	2	3	2	8	21	-	-	-
2014	27	45.27	223.7	105.6	0.60	-	-	10	-	16	-	4	2	2	21	-	1	1

Table S2. Numbers of registered trials and their status: comparison between May and September 2022, according to geographical area and language predominance: LEGEND. Ukr: Ukrine language; Rus: Russian language.

**Oblast**	**Town**	**Main spoken language**	**Areas**	**May**					**September**					
				**Not yet recruiting**	**Recruiting**	**Active, not recruiting**	**Suspended**	**Withdrawn**	**Not yet recruiting**	**Recruiting**	**Active, not recruiting**	**Suspended**	**Withdrawn**	**Terminated**
**Kyiv**	Kyiv	Ukr.	Center	6	20	4	14		4	17	12	14		
	Kodosivka	Ukr.	Center		1		1				2	2		
	Pliuty	Ukr.	Center	1	1				2		1			
	Vyshhorod	Ukr.	Center								1			

Odessa	Odessa	Rus.	South	2	3	1	2		1	3	3	3	1	1
Dnipro	Dnipro	Rus.	South	6	11	1	9		4	9	9	8	1	
	Kryvyi Rih	Rus.	South	1	6	1	4		2	4	3	4		
Mykolaiv	Mykolaiv	Rus.	South	1	1				1	1				
Charkiv	Kharkiv	Rus.	East	5	14	1	12	1	3	12	14	8		
Kirovograd	Kapitanivka	Ukr.	Center		2					1				
Sumy	Sumy	Ukr.	Center	1	6	1			1	4	4			1
Ternopil’	Ternopil’	Ukr.	West		1					1	1			
Vinnytsia	Vinnytsia	Ukr.	West	2	5		2		4	4	2	2		
Volyns’ka	Lukc’s	Ukr.	West	1	5	1	2		1	5	3	3		
Lviv	Lviv	Ukr.	West	1	4		4			4	4	4		
Zaporižžja	Zaporižžja	Ukr.	South	3	4		4		2	4	1	3		3
Kirovohrad	Kirovohrad	Ukr.	Center		3		2			4	1	6	1	
Černivci	Černivci	Ukr.	West				2			3	2	4		
Poltava	Poltava	Ukr.	Center		1		1			1				
	Kremenchuk	Ukr.	Center				1					1		

Žytomyr	Žytomyr	Ukr.	Center		1	2		1		2		
Ivano-Frankivsk	Ivano-Frankivsk	Ukr.	West	1		6			3	6	1	
Zakarpats′ka	Uzhgorod	Ukr.	West	1	3	1		2	3	1		
Cherkasy	Cherkasy	Ukr.	Center		3	3		1	1	2		
Cherson	Cherson	Ukr.	South	1	1	1	1		1		1	
Černihiv	Černihiv	Ukr.	c Center		4	5		1		7	1	9
Chmel′nyc′kyj	Chmel′nyc′kyj	Ukr.	West	2		1	2			1		1

Table S3. Numbers of registered trials and their status: comparison between May and September 2022 according to trial status.

	**Russian language**				**Ukraininan language**			
	**Active**	**Suspended**	**Withdrawn**	**Terminated**	**Active**	**Suspended**	**Withdrawn**	**Terminated**
**May**	54	27	1	0	91	52	0	0
**September**	69	23	2	1	112	58	0	14
	**Centre**				**West**			
**May**	55	29	0	0	27	18	0	

**September**	59	34	2	9	4	21	1	1
	**Est**				**South**			
**May**	32	12	1	0	43	20	0	0
**September**	29	8	0	0	49	18	3	4
